# P-739. Injection-Related Skin Ulcers: a Common and Poorly Addressed Risk Factor for Rehospitalization among People who Inject Drugs in the CHOICE+ Cohort

**DOI:** 10.1093/ofid/ofae631.935

**Published:** 2025-01-29

**Authors:** Edward C Traver, Ishan Kumar Vaish, Habib Omari, Hannah E Flores, Jasmine Stevens, Meghan Derenoncourt, Becky Reece, Alaina Steck, Irene Kuo, Ayako Wendy Fujita, Sumitha Raman, Jillian S Catalanotti, Joseph E Carpenter, Sarah Kattakuzhy, Elana S Rosenthal

**Affiliations:** University of Maryland School of Medicine, Baltimore, MD; University of Maryland Medical School, Baltimore, Maryland; University of Maryland Baltimore, Baltimore, Maryland; University of Maryland, Baltimore, Maryland; University of Maryland School of Medicine, Baltimore, MD; University of Maryland, Baltimore, Baltimore, Maryland; West Virginia University, Morgantown, WV; Emory University, Atlanta, Georgia; George Washington University Milken Institute School of Public Health, Washington, District of Columbia; Emory University School of Medicine, Atlanta, Georgia; George Washington University, Washington, District of Columbia; The George Washington University of Medicine and Health Sciences, Washington, District of Columbia; Emory University School of Medicine, Atlanta, Georgia; Institute for Human Virology (IHV), University of Maryland School of Medicine, Baltimore, Maryland; Institute for Human Virology (IHV), University of Maryland School of Medicine, Baltimore, Maryland

## Abstract

**Background:**

Injection-related skin ulcers (IRSU) are a cause of acute bacterial infection among people who inject drugs (PWID). We aimed to measure the prevalence, characteristics, and risk factors for IRSU, and the risk of readmission, among a cohort of PWID hospitalized for infections.

Table 1

**Figure:**
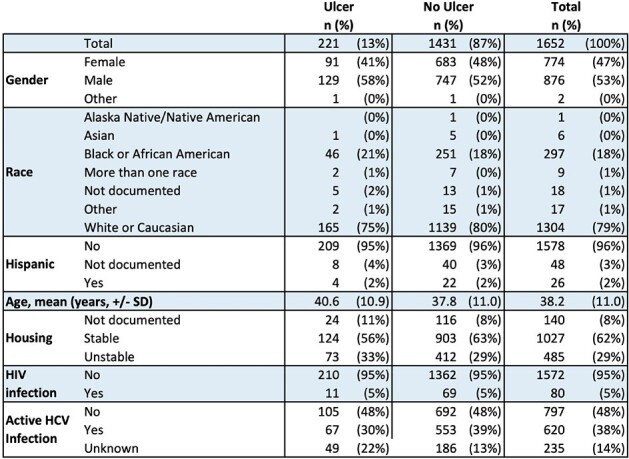

SD, standard deviation.

**Methods:**

CHOICE+ is a multisite retrospective cohort study of adults hospitalized at four healthcare systems with infections due to injection opioid use between 1/1/2018 and 3/31/2022. Data were collected by abstraction of the electronic medical record and were analyzed by chi-square, multivariable logistic regression, and unpaired t-test. Factors statistically significant for IRSU in bivariate analysis were examined by multivariate logistic regression. Detailed data on IRSU were recorded from the Baltimore site.

Table 2

**Figure:**
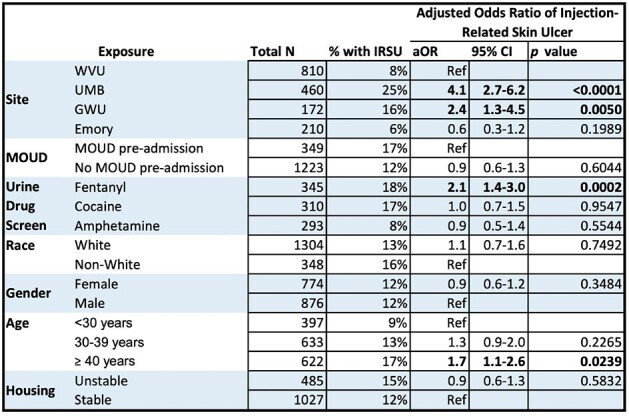

GWU, George Washington University; MOUD, medication for opioid use disorder; UMB, University of Maryland Baltimore; WVU, West Virginia University.

**Results:**

Of 1652 included patients, 221 (13%) had a documented IRSU. Key demographics were similar between those with and without IRSU (Table 1). The odds of IRSU were higher among those who were positive for fentanyl and older age (Table 2). Baseline IRSU was associated with increased odds of readmission (aOR 1.4, p = 0.02) and a greater number of readmissions within 1 year (1.2 v. 0.9, p = 0.01, Figure 1).

Among 116 patients from Baltimore with documented IRSU and detailed data, IRSU was the primary reason for admission in 66%. Xylazine use was not noted in any records. Most patients had < 4 ulcers and ulcers < 10 cm. 13% of patients received sharp debridement and 6% were treated with limb amputation. IRSU was more frequently addressed in the discharge note if IRSU was the primary reason for admission (71% v 50%, p < 0.05). Only 16 (14%) patients were advised or scheduled to attend an outpatient wound care appointment; only 3 (3%) did so within 30 days of discharge (Figure 2).

Figure 1

**Figure:**
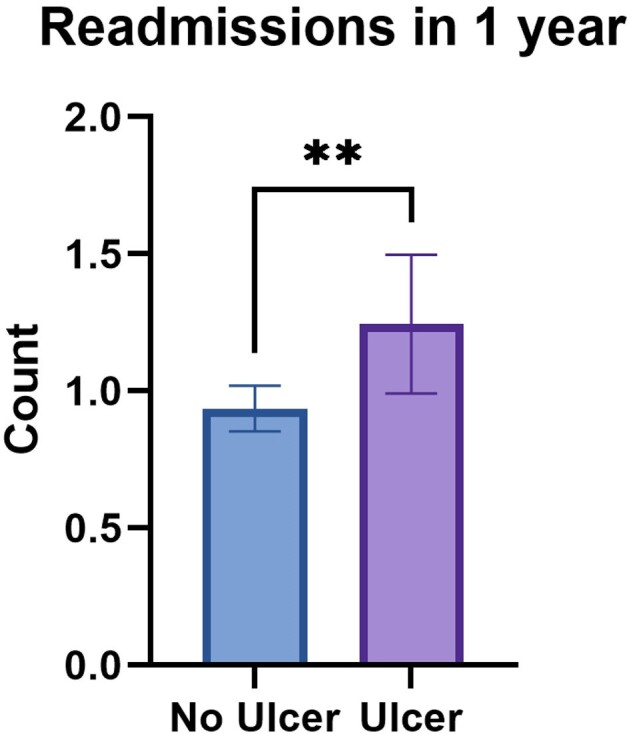

**, p=0.01.

**Conclusion:**

Among PWID hospitalized for infection, IRSU were common, especially among older patients and those testing positive for fentanyl. Xylazine exposure was likely under-assessed. Importantly, IRSU was associated with an increased risk of hospital readmission, but outpatient planning and follow up were rare. Clinicians caring for PWID must address IRSU alongside other infectious diseases, addiction, and harm reduction interventions.

Figure 2

**Figure:**
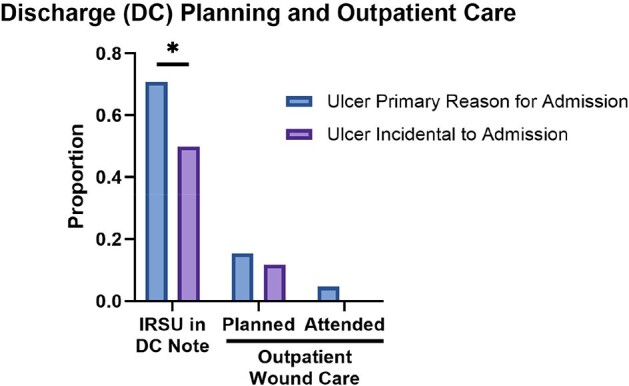

Discharge planning and outpatient care within 30 days of discharge. DC, discharge; *, p=0.048)

**Disclosures:**

**Elana S. Rosenthal, MD**, Gilead Sciences: Grant/Research Support|Merck: Grant/Research Support

